# Working Inside for Smoking Elimination (Project W.I.S.E.) study design and rationale to prevent return to smoking after release from a smoke free prison

**DOI:** 10.1186/1471-2458-11-767

**Published:** 2011-10-05

**Authors:** Jennifer G Clarke, Rosemarie A Martin, LAR Stein, Cheryl E Lopes, Jennifer Mello, Peter Friedmann, Beth Bock

**Affiliations:** 1 Brown University Center for Primary Care and Prevention, Pawtucket, USA; 2 Memorial Hospital of Rhode Island, Pawtucket, USA; 3 Bio Med Alcohol & Addiction, Alpert Medical School of Brown University, Providence, USA; 4 Department of Psychology, University of Rhode Island, Kingston, USA; 5 Rhode Island Department of Corrections, Cranston, USA; 6 Bio-Med Institute of Community Health Promotion, Alpert Medical School of Brown University, Providence, USA; 7 Department of Medicine, Rhode Island Hospital, Providence, USA; 8 Centers for Behavioral and Preventive Medicine, The Miriam Hospital, Providence, USA

## Abstract

**Background:**

Incarcerated individuals suffer disproportionately from the health effects of tobacco smoking due to the high smoking prevalence in this population. In addition there is an over-representation of ethnic and racial minorities, impoverished individuals, and those with mental health and drug addictions in prisons. Increasingly, prisons across the U.S. are becoming smoke free. However, relapse to smoking is common upon release from prison, approaching 90% within a few weeks. No evidence based treatments currently exist to assist individuals to remain abstinent after a period of prolonged, forced abstinence.

**Methods/Design:**

This paper describes the design and rationale of a randomized clinical trial to enhance smoking abstinence rates among individuals following release from a tobacco free prison. The intervention is six weekly sessions of motivational interviewing and cognitive behavioral therapy initiated approximately six weeks prior to release from prison. The control group views six time matched videos weekly starting about six weeks prior to release. Assessments take place in-person 3 weeks after release and then for non-smokers every 3 months up to 12 months. Smoking status is confirmed by urine cotinine.

**Discussion:**

Effective interventions are greatly needed to assist these individuals to remain smoke free and reduce health disparities among this socially and economically challenged group.

**Trial Registration:**

NCT01122589

## Background

Tobacco use contributes to over 400,000 deaths annually [1]. It is a major contributor to both cancer and heart disease risk, and is the leading cause of preventable morbidity, mortality and health expense in the United States [2]. There is an estimated $157 billion in annual health related and economic costs [2]. Quitting smoking reduces the risks of developing smoking related illnesses as well as the morbidity and mortality associated with these illnesses.

In 2009 approximately 46.6 million American adults smoked, an overall prevalence of 20.6% [3]. The prevalence of smoking is much higher among incarcerated populations. Across the U.S. tobacco use among prisoners is approximately three times that of the general population, [4] and in some areas is even higher. For example approximately 80% of the women incarcerated in RI smoked prior to incarceration [5]. Moreover, incarcerated populations suffer disproportionately from health disparities due to tobacco associated illnesses, as minorities, poor, mentally ill and illicit substance using individuals are all overrepresented in correctional facilities. With approximately 9 million Americans passing through correctional institutions annually and an average daily population of over 2 million, there are multiple opportunities to address the smoking cessation needs of this high risk and underserved population [6].

Since the announcement of the negative health consequences of second hand smoke, correctional facilities are increasingly becoming tobacco free. The Rhode Island Department of Corrections (RI DOC) has been tobacco free since February 2003, with no tobacco products allowed anywhere on grounds by inmates or staff. However, the majority of inmates return to smoking as soon as they are released back into the community [7].

Few data are available on relapse prevention interventions delivered after extended periods of forced tobacco abstinence. Medications for smoking cessation are unlikely to be effective as people in this situation have already completed nicotine withdrawal. One approach that has demonstrated efficacy and effectiveness for assisting in smoking cessation and maintenance is cognitive behavioral therapy (CBT) [8,9]. This approach teaches skills that enhance the individual's ability to cope with challenges and remain smoke free. However, CBT is limited in its applicability to the prison setting, in that some skills (delaying time between cigarettes, self-monitoring smoking patterns) cannot be practiced in a smoke free facility. Moreover, skills-based approaches may be moot for individuals who may not be motivated to learn and implement skills. Thus, we chose to enhance a CBT-based intervention with additional sessions of Motivational Interviewing (MI), to enhance interest in remaining smoke free so that skills will be learned and utilized post-release from incarceration [10,11]. This study is designed to evaluate the effects of an Intensive Behavioral Intervention (IBI, which combines MI+CBT) on post-release smoking abstinence rates as well as motivation to remain abstinent among a population of incarcerated men and women who had smoked prior to incarceration and are scheduled to be released.

## Methods/Design

### Design

The study design is a randomized, controlled trial comparing two groups: 1) six sessions of individual MI and CBT counseling (**IBI**) and 2) six general wellness videos (**Control**). Participants are recruited and randomly assigned to either the IBI or time-equivalent control condition eight weeks prior to their release date from a smoke free prison. Those randomized to IBI will receive two MI and four CBT sessions in prison (1 session per week) as well as two brief (approximately 15 minutes) telephone counseling sessions after their release. Assessments are conducted in person at baseline, by phone 24 hours and 7 days after release, and in person at the final three week post-release follow up. This study is approved by the Memorial Hospital of RI Institutional Review Board, the Office for Human Research Protections and the Medical Research Advisory Group at the RI DOC.

### Sample Size Considerations

Sample size was selected to permit analysis of the primary research questions at an alpha of .05 one-tailed and a power level of at least .80. The primary dependent variable used in the power analysis is point-prevalence abstinence (self-report of 7-day abstinence confirmed by cotinine, per Hughes) [12]. Based on three Cochrane Database of Systematic Reviews [13-15] and a very extensive meta-analysis of approximately 8,700 studies that addressed assessment and treatment of tobacco dependence [13,16] we used 23% abstinence as the estimate of abstinence in the intervention Group. We used a conservative estimate of abstinence of 14% for the control group for a sample size requirement of 235 per group. We planned for an interim analysis after 200 participants were recruited as one study found that 97% of inmates return to tobacco use with no treatment by six months following release [17].

### Participant recruitment

Potential participants are recruited by Research Assistants (RAs) in the housing units at the RI DOC sentenced women's facilities and men's medium security facility. RAs will identify themselves as research staff (not RI DOC staff) and inform potential participants that study participation is completely voluntary and does not affect any privileges at the facility including probation or parole status. The planning and research unit of the RI DOC provides a weekly list of all inmates scheduled to be released. RAs review the list for all inmates scheduled to be released within the next eight weeks and call them individually to a private area to be informed about the study. Individuals who are interested in participating are screened for eligibility. Both men and women are eligible to participate if they are 18 years or older, smoked at least ten cigarettes per day prior to incarceration, speak English, and are scheduled to be released within eight weeks of study enrollment. Eligible individuals are given a consent form to review and the RA explains the study. If eligible and willing to participate in the study, the informed consent process is completed (study explained, consent form read to the individual, questions answered and forms signed). The RA provides all participants with an American Heart Association smoking cessation pamphlet, a list of community resources and study contact information. A 60 minute computer assisted questionnaire is administered to all participants at baseline, prior to any intervention activities.

### Procedures

After the baseline assessment is completed participants are randomly assigned to either IBI or Control. Randomization is stratified by gender, the amount of cigarettes smoked in the thirty days prior to incarceration (less than 20 cigarettes a day vs. 20 or more cigarettes a day), and post release smoking plans. Plans to remain tobacco abstinent are assessed with a single question "*Which ONE statement BEST describes your plans for smoking?*" with low levels of planning to remain abstinent including responses "I plan to smoke when I get out of here and I never plan to quit" to "I will probably smoke when I get out of here". High levels of planning to remain abstinent include responses ranging from "I probably won't smoke when I get out of here" to "I have made plans to not smoke when I get out and I will never smoke again." Randomization is concealed and takes place independently of the RAs. IBI counseling and Control video sessions are scheduled weekly, however, if a release date is moved forward then the frequency of sessions or videos is increased such that all six are completed prior to the participant's release.

### Interventions

#### IBI

Intervention sessions are conducted over an approximately six week period prior to release. Participants randomized to IBI receive six counseling sessions, while still incarcerated. In sessions one and six the participant receives MI counseling and in sessions 2-5 the participants receive CBT counseling. Sessions are delivered by RAs who have undergone intensive training with experts in MI and CBT. Supervision is conducted twice monthly to ensure continued adherence and competence with the MI and CBT protocols. Sessions last between 30 and 60 minutes depending on the needs of the participants. The research counselors' therapeutic style and protocol are based on the principles of MI, with a focus on empathy, not arguing, developing discrepancy, self-efficacy, and personal choice[18]. Sections of the MI include developing rapport, exploration of motivation (pros and cons), personalized assessment feedback, and establishing goals. The final MI session mirrors the first session, but adds additional emphasis on re-assessing and increasing motivation to be smoke-free with release date approaching. With participant permission, a quit plan is developed with post-release goals, reasons for goals, specific actions and supports, and possible barriers to goals and solutions. Participants who do not wish to develop goals are thanked for their time, counselors acknowledge that only the participant is able to make a decision regarding his/her commitment to smoking abstinence, and these participants are invited to use the information provided in the counseling sessions in the future if they would like.

The CBT sessions are delivered using a standardized program manual and work sheets to ensure consistent delivery of content. CBT sessions teach smokers to recognize specific environmental and affective events ("*triggers*") that occur prior to smoking and to identify behavioral and cognitive strategies to cope with these triggers. Participants are provided with information on numerous strategies to assist with maintaining smoking abstinence and renewing cessation in case of relapse. These strategies include avoiding trigger situations (e.g., temporarily avoiding drinking coffee), altering these situations (e.g., drinking juice instead of coffee), and engaging in an alternative behavior (e.g., going for a walk, or practicing deep breathing instead of smoking). Additional brief telephone sessions are conducted at approximately 24 hours and 7 days after the individual's release. These sessions included elements of both MI and CBT in an effort to re-evaluate and establish motivation and use of skills after release.

#### Control

Individuals randomized to the Control condition watched a series of videos weekly to match the IBI condition on time spent and frequency of contact. The control videos are between 30 and 60 minutes long and include a variety of health related topics (1. "Managing Chronic Pain," 2. "Managing Migraines," 3. "Managing Respiratory Infections," 4. & 5. "Super Size Me," 6. "Know your Numbers"). To maintain frequency and duration of contact, telephone calls are scheduled for approximately 24 hours and 7 days after release, and are used assessment of smoking status only (no intervention is administered).

#### Quality Control

All intervention sessions are audio recorded and a random 20% are reviewed and scored for intervention content. The MI sessions are scored using the MITI 3.1 [19] for MI compliance, and CBT sessions are independently scored to ensure scheduled content is adequately covered in each session. RAs receive two group supervision sessions a month; one for CBT and one for MI.

### Measures

Participants complete full assessments at baseline and at three week follow up, and brief assessments of smoking status at 24 hours and 7 days post-release. Audio Computer-Assisted Self-Interviews (A-CASI) are used to collect information at baseline and the week three follow up. With A-CASI, survey respondents listen to questions on headphones, see them on a computer screen, and answer them directly on the computer. The RA is available to assist in case of technical difficulties and to answer participant questions. The full assessment battery takes about 60 minutes to complete and includes demographic variables, smoking history and nicotine dependence assessed by the Fagerstrom Test for Nicotine Dependence [20]. Smoking decisional balance is assessed with The Decisional Balance Scale (short form) a six item measure of the pros and cons of smoking [21]. Smoking cessation self-efficacy is assessed using the nine-item short form of the Smoking Situations Confidence questionnaire [22]. Motivation to remain tobacco free is assessed with the Contemplation Ladder, [23] modified to account for the smoke free environment. The four-item Perceived Stress Scale (PSS) [24] is used to document subjective increases in stress. Substance use is evaluated with the Addiction Severity Index (ASI), [25,26] and depressive symptoms with the 10-item Center for Epidemiologic Studies Depression Scale CES-D [27]. Reviews of prison records are done to obtain the number of days between incarceration and release as well as the number of prior incarcerations. At the three week follow up a urine sample is obtained to test for Cotinine, THC (marijuana), cocaine, PCP, opiates, methamphetamines, methadone, amphetamines, barbiturates, benzodiazepines and MDMA ("ecstasy").

A brief questionnaire is administered to assess for changes in motivation to quit, stress and depression. A detailed timeline follow back (TLFB) of the time since release is administered to assess tobacco use as well as drug and alcohol use, living situation and a detailed history the situation leading to the first cigarette if a relapse occurs. For participants who are smoke free at three weeks, monthly phone surveys are completed to assess continuous abstinence and in-person follow ups are scheduled for three and six months post-release to biologically confirm the smoking status.

### Outcomes

The primary outcomes of this study are smoking abstinence rates post release as well as number of days to first cigarette after release. Seven day point-prevalence abstinence will be determined by combining self-reported tobacco use over the prior seven days and urine cotinine assays (below 200 ng/ml versus 200 or above). Participants with self-reported abstinence and cotinine levels below the cut-off will be classified as abstinent. Participants lost to follow-up will be considered non-abstinent. A secondary analysis will examine the effect of length of forced abstinence on quit rates.

### Time plan

The development of this program began in August of 2009 and was finished in February 2010. Participant recruitment started in March 2010 and is ongoing with plans to complete the study in August 2011.

### Planned Analyses

#### Overview

All analyses will be based on intent to treat; participants who receive their assignment to condition will be used in analyses [28]. The primary hypothesis of the study will be tested using generalized estimating equations (GEE) [29]. We will test whether IBI will result in significantly more seven-day point-prevalence compared to Control. We will also analyze three secondary smoking variables: (1) percent days abstinent post release; (2) average number of cigarettes/day; and (3) number of days to first cigarette after release. We will use mixed effects models for the secondary smoking outcomes and will covary the same variables assessed at baseline. We will also examine substance use outcomes by calculating the percents days used other drugs and alcohol from the ASI. We will test the effects of varying lengths of forced abstinence (incarceration) on tobacco quit rates (seven-day point prevalence) using GEE. Our hypothesis is that, regardless of intervention condition, longer incarcerations will be associated with greater quit rates at 3 weeks post release. For this analysis the length of forced abstinence (in days) calculated from first date of incarceration to date of release will be used to predict seven-day point-prevalence.

Further analyses will examine the effects of IBI on smoking outcomes is attributable to potential moderators and if the relationship between IBI and smoking outcome is mediated by relevant variables [30,31] We will test the following potential mediators: education, race/ethnicity, motivation to quit smoking and smoking related illnesses.

In terms of moderators, we expect that IBI will have an especially strong positive effect on smoking outcomes, relative to Control, in the presence of older age, high levels of stress, living with a smoker, homelessness, depression/anxiety disorders, male gender, nicotine dependence, lower SES and social support.

## Discussion

If this intervention is efficacious, it can address the important issue of how to maintain smoking cessation after forced abstinence due to incarceration. Once released from a smoke free environment (incarceration, basic military training, substance abuse treatment or psychiatric facility) the vast majority of smokers immediately start smoking again [7,32-34]. Hence, despite forced abstinence many smokers need interventions to decrease relapse post release. As described above, CBT has demonstrated efficacy/effectiveness; however, skills-based interventions may be less effective for persons unmotivated to implement them. The current literature does not address a combined approach to both a) enhance interest in maintaining cessation and b) learn and implement skills during forced abstinence while incarcerated. This study addresses this gap in the knowledge base. In addition, given the racial/ethnic diversity often found in these settings, it is important to study interventions that may be suitable to this diverse population.

This study is an initial investigation of the efficacy of combined MI/CBT for smoking abstinence after release. While prior studies used longer follow-ups, we chose an abbreviated follow-up because relapse to smoking is rapid after release and this is the first intervention study of its kind conducted in this setting. The short term outcomes of this study will reveal if MI/CBT has the potential to improve smoking cessation rates after release from prison and will provide information to adjust the intervention if needed. The design chosen for this study will allow us to explore potential mediators of the intervention's efficacy so that we may better understand the mechanism(s) by which this intervention may impact maintenance of smoking cessation.

Innovative treatments are needed that address enhancing interest in and skills for maintaining a smoke-free life-style after forced abstinence due to incarceration. This is a highly underserved population in great need. MI has been associated with good outcomes in racially/ethnically diverse samples, [35,36] which is significant for prison settings that reflect high proportions of ethnic and racial minorities. A motivationally based intervention revealed that for smokers not motivated to quit, the intervention led to an odds ratio of quitting of 1.79 over controls and even higher (4.9) for ethnic minorities [32]. Similarly, CBT also demonstrates good intervention effects for racial and ethnic minorities [8,37,38]. Although some meta-analyses have suggested that the effects of MI may not be robust for smoking, [39] others have argued against this [36,40]. However, our use of MI is to enhance interest in change (i.e., maintaining cessation) and in engaging with additional treatment, and both uses have demonstrated efficacy [35,41-43]. Therefore, MI+CBT may prove to be a more efficacious treatment for maintaining smoking abstinence after release from incarceration than either treatment alone. Importantly, this study will afford the opportunity for follow-up after release from incarceration, which is rarely or ever found in the literature with respect to smoking behaviors.

Incarcerated people have higher smoking prevalence than the general population and suffer disproportionately from the health effects of tobacco smoking due over-representation of ethnic and racial minorities, impoverished individuals, and those with mental health and drug addictions [17,44-48]. The incarcerated setting provides a unique opportunity to intervene with this population prior to their release back into the community. Provision of an individually-tailored intervention to this particular population in this specific setting represents a highly innovative and extremely important effort to reach a vulnerable population of smokers.

## Competing interests

The authors declare that they have no competing interests.

## Authors' contributions

JGC conceived of the study, and participated in its design and coordination and helped to draft the manuscript. RAM participated in the design of the study and performed the statistical analysis. LARS participated in the study design and helped to draft the manuscript. CEL participated in the study design and helped to draft the manuscript. JM participated in the study design and helped to draft the manuscript. PF participated in the study design and helped to draft the manuscript. BB participated in the study design and helped to draft the manuscript.

All authors read and approved the final manuscript.

## Pre-publication history

The pre-publication history for this paper can be accessed here:

http://www.biomedcentral.com/1471-2458/11/767/prepub
